# The Superintegron Integrase and the Cassette Promoters Are Co-Regulated in *Vibrio cholerae*

**DOI:** 10.1371/journal.pone.0091194

**Published:** 2014-03-10

**Authors:** Evelyne Krin, Guillaume Cambray, Didier Mazel

**Affiliations:** 1 Institut Pasteur, Unité de Plasticité du Génome Bactérien, Département Génomes et Génétique, Paris, France; 2 CNRS, UMR 3525, Paris, France; East Carolina University School of Medicine, United States of America

## Abstract

Chromosome 2 of *Vibrio cholerae* carries a chromosomal superintegron, composed of an integrase, a cassette integration site (*attI*) and an array of mostly promoterless gene cassettes. We determined the precise location of the promoter, Pc, which drives the transcription of the first cassettes of the *V. cholerae* superintegron. We found that cassette mRNA starts 65 bp upstream of the *attI* site, so that the inversely oriented promoters Pc and Pint (integrase promoter) partly overlap, allowing for their potential co-regulation. Pint was previously shown to be induced during the SOS response and is further controlled by the catabolite repression cAMP-CRP complex. We found that cassette expression from Pc was also controlled by the cAMP-CRP complex, but is not part of the SOS regulon. Pint and Pc promoters were both found to be induced in rich medium, at high temperature, high salinity and at the end of exponential growth phase, although at very different levels and independently of sigma factor RpoS. All these results show that expression from the integrase and cassette promoters can take place at the same time, thus leading to coordinated excisions and integrations within the superintegron and potentially coupling cassette shuffling to immediate selective advantage.

## Introduction

**Figure 1:**
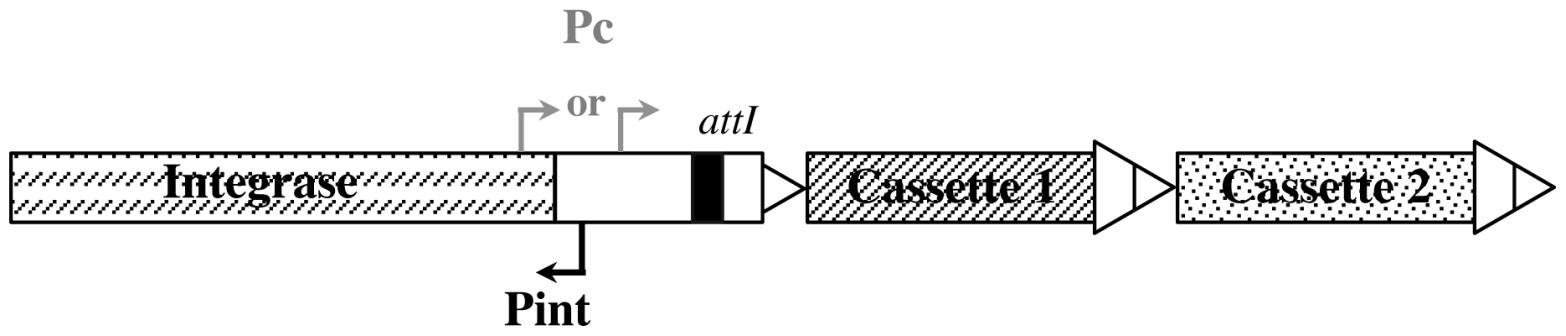
Schematic organization of an integron platform. Pc and Pint are the cassette array and integrase promoters, respectively. Pc can be located in one or another position. *attI* is the primary recombination site for integrase. Double triangles correspond to *attC* sites.

*Vibrio cholerae*, a marine bacterium, is the causative agent of cholera in humans, a severe diarrheal disease often fatal by dehydration. Its genome is characterized by the presence of two chromosomes: a large one which contains the vast majority of genes essential for cell functions and pathogenicity, and a small one which contains a large number of genes of hypothetical function and of exogenous origins [1]. The small chromosome also carries a gene capture system, found in all *Vibrio* species, called a superintegron (SI) [2]–[4]. All characterized integrons are composed of a stable platform, which contains the functional elements required for the system operation, associated with a variable array of individual gene cassettes which may encode accessory functions [5]. The integron platform is defined by an integrase encoding gene, *intI*, a primary recombination site, *attI*, and a strong promoter, Pc, located upstream of *attI*, either in the non coding sequence upstream of *intI* or in *intI* itself (Figure 1). The gene cassettes are usually composed of a single coding sequence associated with a recombination site, *attC*, which is the target of *IntI*-mediated site-specific recombination with *attI*. In *V. cholerae*, the SI gathers hundreds of genes cassettes (179 in N16961 strain [6]), carrying the highly conserved *V. cholerae* specific *attC* sites, termed VCR (*Vibrio cholerae* repeats), in a single cassette array. While most of the cassettes found in the *Vibrio* chromosomal integrons have unknown functions, the few that were characterized comprise phage-related proteins, toxin/antitoxin systems, acetyltransferases, sulfate-binding proteins, lipases, polysaccharide biosynthesis proteins, dNTP pyrophosphohydrolases, DNA modifying enzymes and virulence factors [3], [7]–[11]. It was thus suggested that cassette arrays carry important functions that could mediate adaptation and interactions with the environment [5], [12].

During the course of its life cycle, *V. cholerae* alternates between various environmental growth conditions: freshwater - which is regarded as its reservoir - brackish water, planktonic growth, biofilm formation on crustacean shells in the sea and transit through the “gastric acidity barrier” before colonizing the intestinal tract during human infection [13]. Thus, understanding cassette expression in these diverse conditions is necessary to grasp the adaptive potential of the integron system.

Several cassettes of large chromosomal integrons, such as those carrying toxin-antitoxin gene pairs, have been found to carry their own promoter [3], [10], [11]. In addition, occasionally functional promoters can be created by the junction of a given *attC* site and the adjacent cassette, as it has been described in the chromosomal integron of *Vibrio* sp. DAT722. This can explain how a portion of the cassette array is transcribed [14] and how it may possibly contribute to the phenotype of this strain [15]. However, most of the resistance cassettes found in mobile integrons involved in antibiotic resistance development and spread lack a promoter [16]. Instead, their expression relies on a strong promoter located upstream of the recombination point, either in the *attI* itself, as in class 2 integrons [17], [18] or in the *intI* gene, as in class 1 and 3 integrons [19]–[22]. In *V. cholerae* most uncharacterized cassettes carry an open reading frame with a potential start codon located very close to the recombination site, leaving no space for a promoter. As for the characterized cassettes, their transcription relies on the Pc promoter carried in the platform. The Vch IntIA integrase allows cassette excision through recombination between two VCR sites and cassette integration through *attI* x VCR recombination [23]. In chromosomal integrons, such as the *V. cholerae* SI, no Pc cassette array promoter has yet been characterized and the question of how cassette genes are expressed remains unanswered. However, it was recently found that the *catB9* cassette [24] confers resistance to chloramphenicol when relocated from its original remote and silent location to the second position of the array, [25], suggesting that a strong Pc promoter is present in the SI platform.

On the other hand, the Pint promoter, which controls the expression of the *V. cholerae* integron integrase, has been characterized and shown to be directly controlled by LexA and induced during the SOS response, and also by the cAMP-CRP complex [26], [27]. SOS is induced when an abnormally high level of single-stranded DNA (ssDNA) is present in the cell, for instance as a consequence of DNA damage or during horizontal gene transfer. This allows the formation of RecA-ssDNA nucleofilaments which trigger the LexA repressor's autocatalytic activity [28]. Once cleaved, LexA is unable to bind SOS-regulated promoters and so allows the expression of the SOS regulon [28]. The cAMP-CRP complex is a global regulator, composed of the Catabolite gene activator and cyclic adenosine monophosphate (cAMP). It is involved in the regulation —repression as well as activation— of a vast number of genes (e.g. adaptation to growth conditions under limited nutrient supplies) through sensing a global signal (e.g. glucose starvation, which is reflected by the intracellular concentration of cAMP) [29], [30]. This dual regulation of *intIA* by LexA and cAMP-CRP occurs via specific regulatory boxes located in the superintegron integrase (*intIA*) promoter region [26], [27]. As this region is located upstream the cassette array, these two regulatory pathways might also control expression from the cassette array promoter.

In order to shed light on the conditions which regulate the expression of the *V. cholerae* superintegron cassettes, we first characterized the mRNA start site and determined the location of the cassette promoter (Pc). We then explored how various growth conditions impact the Pc and Pint activities, in order to define favorable environmental factors that increase the expression of the cassette array and/or of the integrase. The role of the SOS response and of the cAMP-CRP complex in cassette array expression was also investigated.

## Materials and Methods

### Bacterial strains, plasmids, and growth conditions

Bacterial strains and plasmids used in this study are listed in Table 1. Primers are listed in Table 2. *lacZ, crp* or *recA* deletions were introduced in the *V. cholerae* N16961 chromosome by homologous recombination as previously described [31]. Strains were grown in Marine Broth (Roth) (NaCl 19.4 g/l: 332 mM), in Luria Broth (BD) with various NaCl concentrations (0 g/l, 5 g/l (85 mM, usual LB concentration) or 19.4 g/l high concentration), in 1% Bacto-Tryptone (BD) with 19.4 g/l NaCl and 1% glucose or succinate, or in M63 minimal medium (supplemented with 0.2% glucose, 0.1% casamino acids, 100 µg/ml serine and 10 µg/ml vitamin B1), at 29, 37 or 42°C. When required, antibiotics were added at following concentrations: kanamycin, 25 µg/ml; spectinomycin, 50 µg/ml. Induction of the SOS response was performed in Marine Broth by the addition of 200 ng/ml mitomycin C, as previously described [26].

**Table 1:** Bacterial strains and plasmids.

Strain or plasmid	Identified number	Genotype	Source
**Strains**			
*V.cholerae* N16961	ω7805	*Vibrio cholerae* El Tor non transformable strain	Laboratory collection
N16961 Δ*lacZ*	ω4851	Deletion of *lacZ* from strain 7805	[26]
N16961 Δ*lacZ intIA:lacZ*	ω7450	4851 with *intIA* reporter insertion	[26]
N16961 Δ*lacZ Pc:lacZ*	ω8320	4851 with *p cassette array* reporter insertion with plasmid p7476	This study
N16961 Δ*lacZ Δcrp:spec Pc:lacZ*	ω9369	8320 with *crp* deletion using plasmid p8348	This study
N16961 Δ*lacZ Pc:lacZ CRP binding site mutated*	ωA145	8320 with mutation of CRP binding site using plasmid pA089	This study
N16961 Δ*lacZ Pc:lacZ LexA binding site mutated*	ω9407	8320 with mutation of LexA binding site using plasmid p9327	This study
N16961 Δ*lacZ ΔrpoS Pc:lacZ*	ωB997	8320 with *rpoS* deletion using plasmid pB410	This study
N16961 Δ*lacZ intIA:lacZ* lexAind-	ω7286	4851 with *intIA* reporter insertion with lexAind- mutation	[26]
N16961 Δ*lacZ recN:lacZ*	ω7453	4851 with *recN* reporter insertion	[26]
**Plasmids**			
p4730		*pSW23T-aph [Cm Km]*	[26]
p7476		p4730 with *p cassette arrays*:*lacZ* fusion	This study
p8348		*crp* deletion vector derived from pSW7848 with insertion of *aadA7* between forward and reverse regions of the *V. cholerae crp* gene	[27]
pA089		P7476 with CRP binding site mutated: tgg**TGTGA**tgctcaacatactg -> tgg**CCCTG**tgctcaacatactg	This study
p9327		P7476 with LexA binding site mutated: T**G**GTTATTTGTA**C**AGT**A**T-> T**C**GTTATTTGTA**G**AGTT	This study
pB410		*rpoS* deletion vector derived from pSW7848 with forward and reverse regions of the *V. cholerae rpoS* gene	This study

**Table 2:** Primers.

Primer name	Sequence 5′-3′	Reference
16Srt3	GTAAGGGCCATGATGACTTG	[50]
16srt5	TCAGCTCGTGTTGTGAAATG	[50]
2385	GTCTATTAATTAGATAGCGGTAGCC	This study
2386	ACAAGAAGTTGTTCTAAGGGATTGA	This study
Elrork7_1Fw	AAGAATCCGAGAAAGGGGTGTTTTTAGAAT	This study
Elrork7_1Rw	TTTTGGATCCTCCTCTAACATTAGCAAGAAAGCGCC	This study
GSP1	ACAGATGAAACGCCGAGTTA	This study
GSP2	CGAGTTAACGCCATCAAAAAT	This study
Crpmut5	TATAAGCTGAGATTTTTGAATGGCCCTGTGCTCAACATACTGATTTAGAA	This study
Crpmut3	TTCTAAATCAGTATGTTGAGCACAGGGCCATTCAAAAATCTCAGCTTATA	This study
24	TAAAAACAACCAACAAATACTCTACAAATAACGAGTTAAATATTCAGTGAGAACT	[26]
25	AGTTCTCACTGAATATTTAACTCGTTATTTGTAGAGTATTTGTTGGTTGTTTTTA	[26]

### RNA preparation

Total RNA was purified as previously described [32] but genomic DNA was removed using 2 µl Turbo DNase (AMBION) for 30 min at 37°C instead of the DNA-free DNase treatment.

### Cassette array 5′ RACE-PCR

5′ RACE assay was performed with 2 µg total RNA isolated from strainω8320, using the standard protocol of the SMARTER RACE cDNA amplification kit (Clontech) and primer GSP1 (Table 2). PCR was then performed using primers UPM and GSP2 and the PCR extender kit (5′PRIME). The purified PCR amplification product was then sequenced.

### Construction of the expression reporter strains

Strains were constructed as previously described [26] using 500-base pair homologous regions upstream the site of transcriptional fusion with *lacZ,* using primer pair Elrork7_1Fw/Elrork7_1Rw, and inserted into EcoRI and BamHI sites of p4730 giving rise to p7476. pA089 was used to create the CRP binding site mutant and p9327 for the Pint LexA box mutant. Overlapping sequences were realized to confirm correct fusion insertion in the different mutant strains context. *crp* deletion was introduced in the chromosome of *V. cholerae* by homologous recombination using plasmid p8348, as previously described [26], [27]. *rpoS* deletion was introduced using the same protocol and plasmid pB410.

### LexA and cAMP-CRP complex binding site mutagenesis

Plasmids p9327 (LexA box mutant) and pA089 (cAMP-CRP complex box mutant) were constructed by amplifying the complete plasmid p7476 using Herculase (Stratagene) and primer pairs 24/25 and crpmut5/crpmut3, respectively. After column purification, the amplification product was digested by DpnI to remove the native p7476 and then transformed into *E. coli*. Mutations were confirmed by sequencing.

### β-galactosidase assays

β-galactosidase activities were determined by the method of Miller [33] at 37°C. The assays were performed at least twice on independent cultures. Activities are expressed in U Miller/mg dry weight (U/dry weight in figures and text).

## Results

### Determination of the transcription start site of the SI cassette array

**Figure 2:**
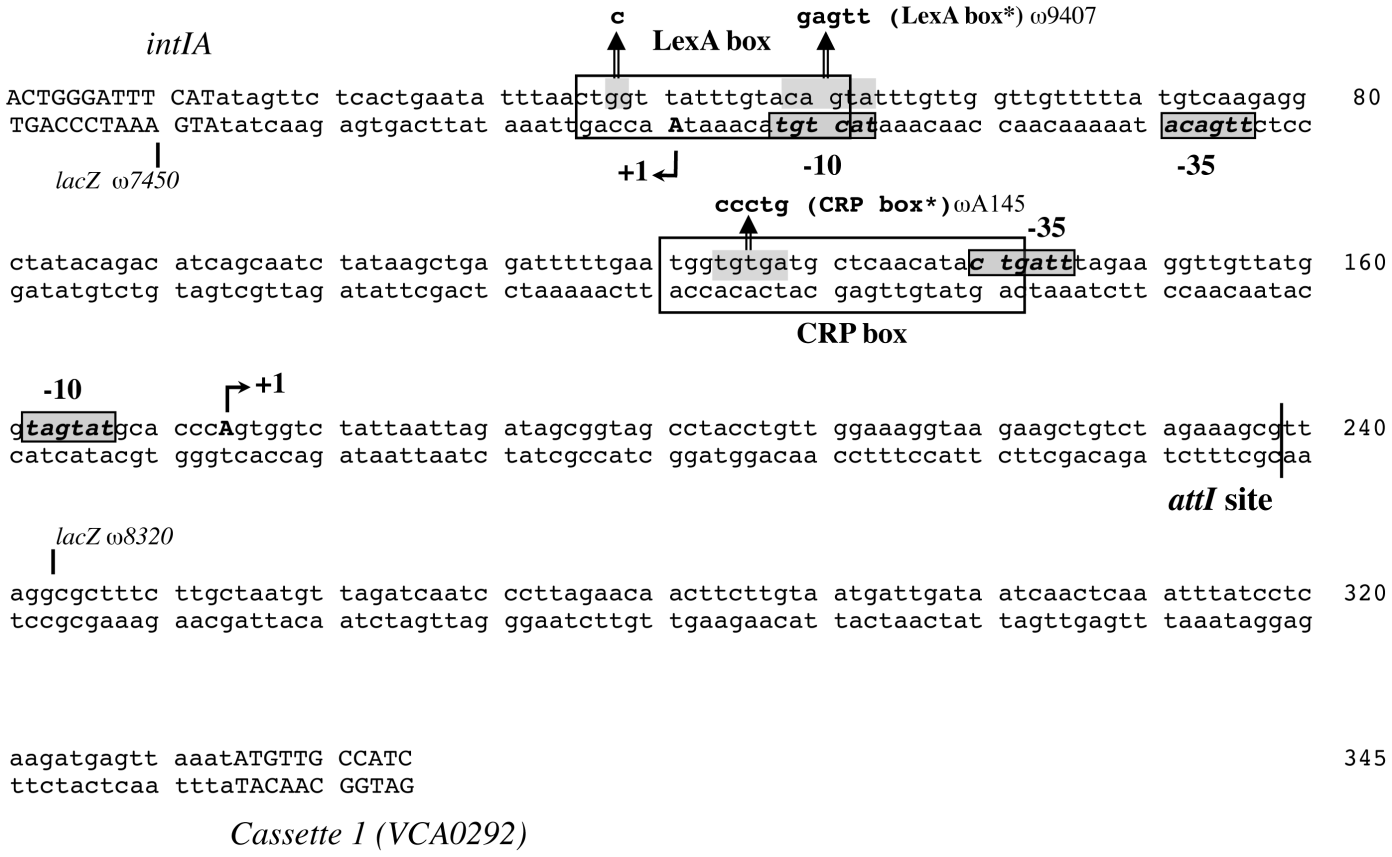
Regulatory region of *intIA* and cassette array and identification of cassette array transcriptional start. The transcriptional start sites (+1) are indicated by arrow and capital and bold letters. The -10 and -35 promoters boxes are shown in grey with bold characters. The coding sequences are indicated with capital letters. Positions of *lacZ* transcriptional fusions are notified by a line. The CRP and LexA boxes mutations are indicated with an arrow and grey boxes (CRP box* and LexA box*). The *attI* recombination point is indicated by a vertical bar.

In the integrons studied to date, the promoter driving the expression of the cassette genes is typically located either in the *intI* gene or in the region between the *attI* recombination site and the *intI* translational start site [17], [19], [21], [22]. However, it remained to be characterized in *V. cholerae*. Determining the position of the transcriptional start site is crucial to study the expression of the cassette array in the *V. cholerae* superintegron. Using a 5′RACE-PCR assay, we located the cassette mRNA start 65 bp upstream of the *attI* recombination point and 132 bp upstream the *intIA* transcription start site (Figure 2). Two hexanucleotide sequences with good match to the -10 and -35 boxes consensus of sigma 70 promoter were identified at proper distance (Figure 2). Thus, considering the Pc localization upstream of the *intIA* promoter there would not be any transcription interference through the antisense pairing of the two corresponding mRNAs, as described in class 1 integrons where Pc is located within the integrase coding sequence [34].

### Expression of the cassette array and of the superintegron integrase depends on growth conditions

**Figure 3:**
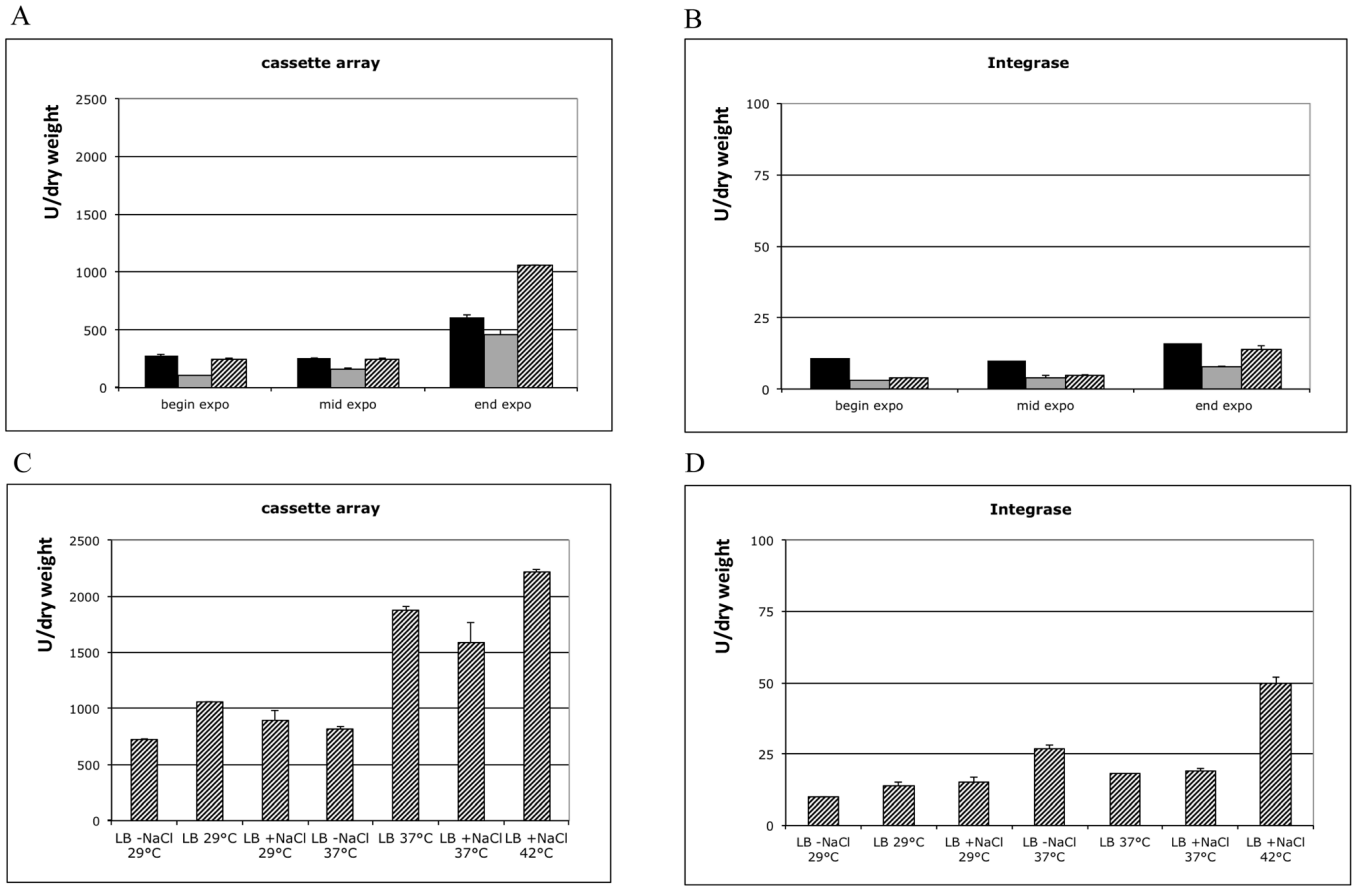
*intIA* and cassette array expression during growth and according to growth conditions. β-galactosidase assays are performed with strains ω8320 (promoter cassette array:*lacZ*) (A and C) and ω7450 (integrase:*lacZ*) (B and D). In A and B, growth was performed at 29°C in M63 glucose casamino serine vitamin B1 (black), in Marine broth (grey) and in classical LB (striped). “expo” means exponential growth. In C and D, growth was performed in LB without NaCl, (-NaCl), with classical concentration (LB) or with high NaCl concentration (+ NaCl) and samples are harvested at the end of exponential growth.

During the course of its lifecycle *V. cholerae* is exposed to environments in which nutrient availability, salt concentration and temperature extensively vary: e.g. freshwater systems (poor in nutrients, low salinity), marine environment (poor in nutrients, high salinity) and the human gastrointestinal tract (rich in nutrients, high salinity and temperature). To investigate the role of these parameters on the expression of the cassette array and of the *intIA* gene, we constructed *lacZ* transcriptional fusions of both Pc and Pint promoters (Figure 2, strain ω8320 and ω7450 respectively). We then studied their activity in culture conditions that simulate the various environments to which *V. cholerae* is naturally exposed: M63 (minimal medium), marine broth (high salt concentration) and LB broth (rich medium) at 29°C (Figure 3A and B). We found that the two promoters are active along the different growth phases, with a maximum at the end of exponential phase, in all media (1.6 to 2.8-fold increase for Pint and 2.4- to 4.4-fold increase for Pc, in comparison to mid-exponential growth phase, in the different culture conditions) (Figure 3A and B). In all the conditions tested, the expression levels measured with the Pint-*lacZ* fusion were always much lower than the ones obtained with the Pc-*lacZ* fusion (25- to 76-fold). The two promoters were generally less active in marine broth while the highest expression levels were observed in rich medium for Pc and in both M63 and LB broth for Pint (Figure 3A and B).

As the maximal Pc driven cassette expression level was measured at the end of exponential phase, this specific period was chosen to assay the impact of temperature and salinity in LB medium, as these parameters vary in different environments encountered by *V. cholerae* (e.g. from brackish water to human intestine). *intIA* expression was found to be 2.7-fold higher at 37°C than at 29°C and, in this condition, its expression was favoured by the absence of NaCl (Figure 3D). However, at 29°C, NaCl induces 1.5-fold the expression of the integrase (Figure 3 D). Pc was shown to be 1.8-fold more active at higher temperatures (37°C vs 29°C) in presence of NaCl (Figure 3C). *V. cholerae* is known to grow at 42°C: this property is used to isolate *V. cholerae* from mixed *Vibrio* populations in invertebrates [35]. We determined the transcription levels from the Pc and Pint promoters at 42°C with a high level of salinity and found respectively a 2.5-fold and 3.3-fold increase in their activity compared to at 29°C (Figure 3C and D), confirming their induction by an increase in temperature. The integrase was found to be more expressed in rich medium (50 U/dry weight) than in minimal medium (40 U/dry weight) at 42°C. Then, the maximal levels of expression from Pint and Pc were obtained in the same condition: LB at a high NaCl concentration, at 42°C. Thus, even if Pc and Pint show different expression levels in a few conditions, they in general show coordinated expression for the cassettes proximal to Pc and for the integrase.

### The SOS response does not control the Pc driven expression of gene cassette

The LexA box located inside the *intIA* promoter might be involved in the Pc driven cassette expression regulation since it is located ≈125 bp upstream of the cassette transcription start site. Indeed, examples of LexA control trough binding to sites remotely located have been previously described [36].

**Figure 4:**
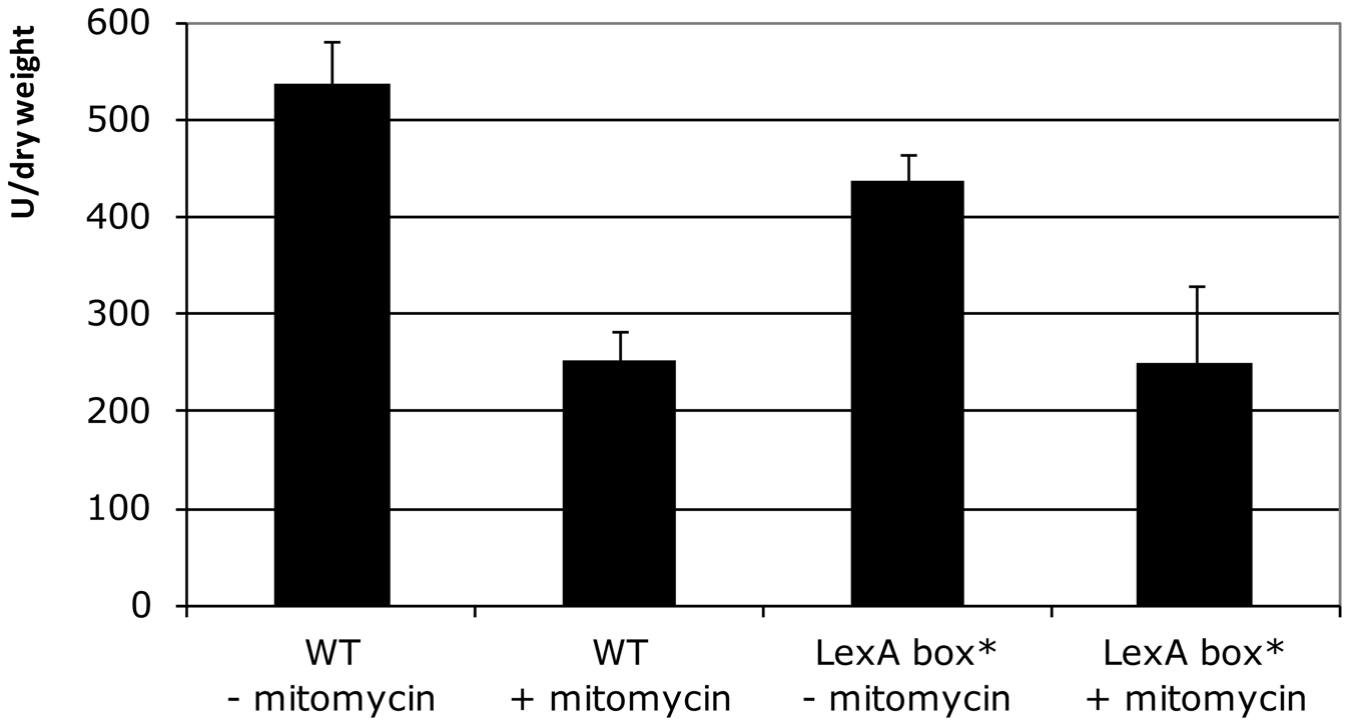
Cassette promoter regulation by SOS response. β-galactosidase assays are performed with strains ω8320 (WT) and ω9407 (LexA box*) after mitomycin C addition (+) or not (-).

We performed β-galactosidase assays in presence and absence of mitomycin C, a DNA-damaging agent known to induce the SOS response in *V. cholerae* [37], to explore this possibility. In wild type strain ω8320 (Pc-lacZ) the addition of mitomycin C led to a 2.1 fold decrease in cassette array expression (Figure 4). Thus, expression from Pc is not induced by SOS. In order to check if the decreased expression by mitomycin C is specific to Pc and related to the LexA box, the same experiment was performed in a LexA box mutant ω9407 (LexA box*) which prevents LexA binding and thus alleviates repression of the promoter by LexA [26], [27]. Mitomycin C led to a similar decrease of expression in this mutant (Figure 4), suggesting that this reduction is due to a change in the general transcription pattern of the cell, when the SOS response is induced. Hence, in contrast with the Pint promoter, the Pc cassette array promoter expression is not induced by the SOS response and does thus not belong to the SOS regulon.

### The Pint induction by temperature is SOS independent

**Figure 5:**
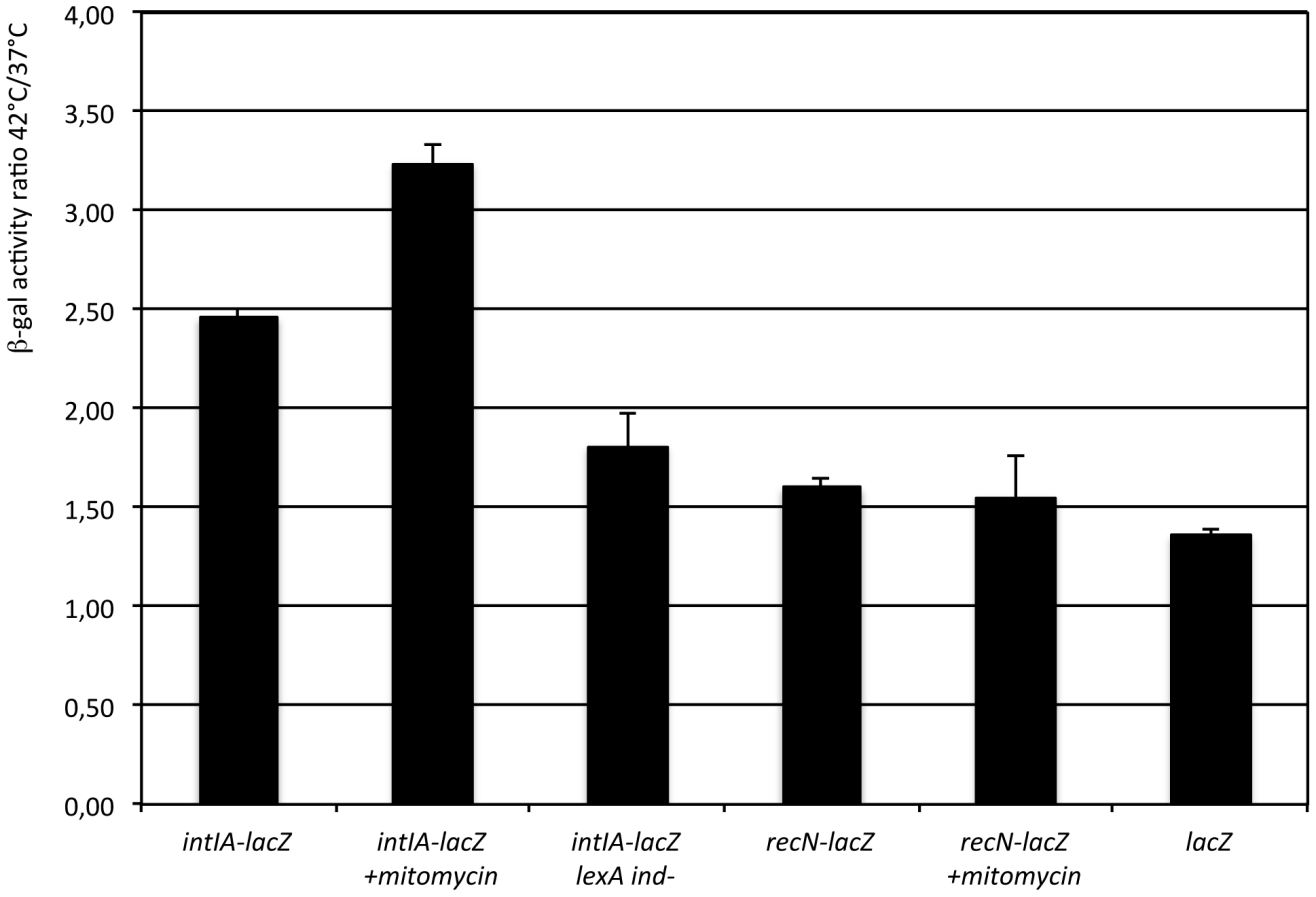
Impact of temperature and SOS response on gene expression. β-galactosidase assays are performed with strains ω7450 (*intIA-lacZ*), ω7286 (*intIA-lacZ lexAind-*), ω7453 (*recN-lacZ*) and N16961 (*lacZ*) after mitomycin C addition (+) or not, harvested at the end of exponential growth.

*intIA* expression is activated by the SOS response induction, which leads to the cleavage of the LexA repressor, preventing its binding to the LexA box located inside the *intIA* promoter and allowing *intIA* transcription [26], [27]. High temperature could increase LexA cleavage in *E. coli* [38] and, as we found that the integrase expression was induced by high temperature, we investigated if the activation of Pint at high temperature was reflecting a lower stability of LexA at 42°C compared to 37°C. Comparison of the ratio of *intIA* expression at 42°C and at 37°C in wild type strain ω7450, showed that expression from Pint is induced 2.5- fold at 42°C compared to at 37°C (Figure 5). This induction ratio is slightly increased in conditions of SOS induction by the addition of mitomycin C at both temperatures, and is reduced when the SOS response is blocked (lexA ind- mutant ω7286, a non inducible LexA derivative) (Figure 5). To clarify the role of a potential higher LexA instability at 42°C, we tested the expression of another SOS regulon gene, *recN* (*recN-lacZ* ω7453) and found a moderate 1.5 fold induction at 42°C compared to 37°C, identical in both mitomycin C induced SOS response or no induction conditions (Figure 5). This suggests that regulation by the temperature is likely not related to the stability of LexA. Furthermore, the ratio of the expression of *recN* at 42°C and at 37°C remains lower than that of *intIA* and similar to that of the *V. cholerae lacZ* gene which is not under the control of the SOS response (Figure 5). Thus *intIA* seems to be specifically regulated by temperature, and this effect is even higher when SOS is induced, by mitomycin C, when there is no LexA to hamper transcription from this promoter (Figure 5). LexA is a strong Pint repressor, and likely its major regulator, leading to a reduced influence of other regulators when it is present.

### Pc is activated by the cAMP-CRP complex

It was recently demonstrated that the cAMP-CRP complex increases *intIA* expression by direct binding upstream of the *intIA* transcription start site [27]. This CRP box is centered at −41.5 bp from the cassette array transcriptional start site and overlaps its -35 region, as a typical Class II activator [30]. This suggested a potential activator role for the cAMP-CRP complex on the Pc promoter regulation.

**Figure 6:**
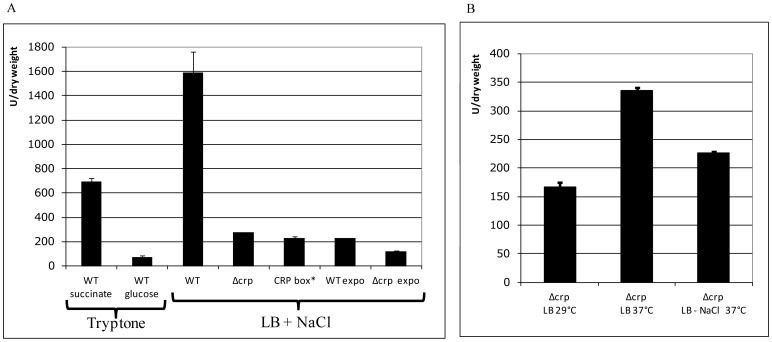
Cassette array regulation by cAMP-CRP complex. β-galactosidase assays are performed with strains ω8320 (WT), ω9369 (Δ*crp*) and ωA145 (CRP box*). All strains were harvested at the beginning of the stationary phase, except for “expo”, harvested at mid exponential phase growth. Bacteria were grown in Tryptone medium (Tryptone) or in LB, with high NaCl concentration (LB + NaCl) or without NaCl (LB - NaCl).

To explore this hypothesis, we performed β-galactosidase assays using strain ω8320 (Pc-*lacZ*), grown until the end of exponential phase in Bacto-Tryptone medium supplemented with 1% glucose or succinate, as previously described [29]. Glucose induces catabolite repression and lowers the cAMP level, while succinate leads to a high cAMP level, allowing cAMP-CRP complex formation and regulation of its associated genes [29], [30]. In presence of glucose, the cassette array expression was 9-fold lower than in presence of succinate. This suggested that the cAMP-CRP complex activates expression of the cassette array (Figure 6).

To confirm this result, a Δ*crp* strain was constructed (strain ω9369), and the *lacZ* fusion expression level was compared with those obtained in the wild type context. Deletion of *crp* led to a 6-fold decrease of the cassette array expression at the end of exponential phase in LB with high concentration of NaCl (Figure 6A), confirming the activator role of the cAMP-CRP complex. We mutated the CRP box located upstream the cassette mRNA start site and overlapping the Pc -35 box to see if it was responsible for the regulatory effect observed (CRP box*, strain ωA145) (Figure 2). This mutation was designed to prevent cAMP-CRP complex binding without affecting binding of the RNA polymerase, as previously described in *Vibrio* [39]. The level of expression of the first cassette in this context was similar to that in the Δ*crp* strain (Figure 6A), demonstrating the direct regulation of Pc by cAMP-CRP through its binding to this CRP box.

As the cAMP-CRP complex is a known global regulator involved in many pathways, we investigated its implication in the activation of the expression of the first cassette at the entry into the stationary phase, and by high temperature and/or high salinity. Deletion of *crp* only led to a 2-fold decrease during the exponential growth phase, in comparison to the 6-fold difference obtained at the beginning of stationary phase (see above, Figure 6A), suggesting a cAMP-CRP complex-dependent regulation at this stage. In addition, in the absence of *crp*, the activation by temperature and salinity remained almost identical to the one observed in the wild type strain (Figure 6B), suggesting a cAMP-CRP complex-independent regulation in these conditions.

Cholera toxin is known to induce the host adenylate cyclase activity and, as a consequence, increase the intracellular cAMP levels in the intestinal cells [40] and subsequently in the intestinal mucosa [41], [42]. Therefore, we investigated if a high extracellular cAMP concentration in the culture medium would induce the integron promoters. The transcription levels observed with a high extracellular cAMP concentration (3 mM) remained identical to those measured in its absence (data not shown), suggesting that its uptake in *V. cholerae* is not efficient enough to increase cAMP-CRP complex concentration and activate expression from the integron platform. In conclusion, cAMP-CRP complex induces both the superintegron integrase and the cassette array expression, via binding to the same CRP box, independently of the extracellular cAMP level.

### The Pc induction by the entry into stationary phase, rich medium, high temperature and high NaCl level is independent from sigma factor RpoS

As the cAMP-CRP complex role only explains the Pc regulation by the entry in stationary phase, we investigated the involvement of RpoS in all the Pc regulations. *rpoS* encodes sigma factor 38, which plays a key role in the response to various environmental changes or stresses such as hyperosmolarity or carbon starvation during stationary phase [43]. To determine its involvement in activating the expression of the first cassette, we compared the *lacZ* reporter expression levels in wild type and *rpoS* deficient (ωB997) strains grown in LB medium, with high level of NaCl, during the entry in stationary phase at 42°C. No significant difference was observed (2216 vs 1855 U/dry weight: 1.17-fold), suggesting that RpoS has no direct or indirect role in the activation of the Pc promoter in these conditions.

## Discussion

We characterized the *V. cholerae* N16961 superintegron cassette array promoter, Pc, which insures the expression of the first cassettes of the array, and found that it is located upstream of the superintegron primary recombination site *attI* (Figure 2). This location is similar to the one found in class 2 integrons [17] and differs from the class 1 integron platform organization [19]–[21], where the cassette promoter resides within the integrase coding sequence. This configuration allows the co-expression from both the superintegron integrase and cassette array promoters (Pint and Pc), at the same time, in contrast with what is observed for class 1 integrons [34]. The DNA region between *intIA* and the *attI* recombination site (containing both promoters, Pint and Pc) is highly conserved in all sequenced *V. cholerae* genomes and similar regulation mechanisms and expression conditions can be expected for the SI integrase and the cassettes of all *V. cholerae* strains. In addition, both the regulatory LexA and CRP boxes, as well as the Pc and Pint sequences are also conserved in the *V. cholerae* closest relative, *Vibrio mimicus*, and it is likely that similar regulatory mechanisms are at play for both promoters in this species (Figure S1).

In this work, we also showed that although the *V. cholerae* N16961 Pc promoter is active in all tested conditions (marine, poor and rich media, with medium (85 mM), high (332 mM) or no NaCl, from 29°C to 42°C), the cassette expression reaching its maximal level at the beginning of the stationary phase (Figure 3). In parallel, although at a much lower level, the superintegron integrase is also expressed in all growth conditions tested and induced during entry in stationary growth phase (Figure 3). The low level of expression and the late growth phase induction of Pint may likely be explained by its regulation by LexA, which is not the case for Pc. Indeed, it has been shown that the SOS response is induced at the entry in stationary phase, in *E. coli*, as a result of LexA degradation [44]. Our results suggest that this may also be the case in *V. cholerae*. Nevertheless, the Pint activation by temperature is independent from a potential increase of LexA cleavage (Figure 4).

We found that both Pc and Pint are highly induced in rich medium, at high temperature and at a high NaCl concentration (Figure 3). These three conditions are combined inside the human intestine, where *V. cholerae* is exposed to a nutrient rich environment. Moreover, *V. cholerae* encounters various stressful conditions during its transition from the marine environment to its host's gastrointestinal tract, such as increased temperature. After intestine invasion, bacteria stop swimming and start colonizing the epithelial layer. Concomitantly, they produce the cholera toxin, which finally induces the activity of the adenylate cyclase, leading to elevated intracellular concentrations of cAMP in the intestinal cells. This causes chloride selective channels to open and Cl^−^ ions are released in the intestinal lumen. In parallel, Na+ ions are also secreted into the small intestine [40]. As a consequence, after cholera toxin production [45], NaCl reaches high concentrations inside the intestine (> 300 mM) [46]. Thus, this coincides with the highest expression of the Pc proximal cassettes, and one can imagine that this gives a chance for the massively excreted bacteria to be better fit for the external unpredictable environment in which they will be released. However, although sigma factor 38 (RpoS) plays a major role in the adaptation to environmental variations [43], it is not involved in the regulation of the expression from the integron platform, suggesting than there is one or more other regulators sensing temperature and/or osmolarity increases to induce the integron platform promoters.

cAMP also plays a crucial role in the life cycle of *V. cholerae* by its association with CRP, to form the cAMP-CRP regulator complex, which affects the expression of genes required for its survival in the human host and the environment [47]. Indeed, the cAMP-CRP complex represses the expression of the cholera toxin and of the toxin co-regulated pilus in low nutrient environments (outside the host) and, in contrast, in rich media such as the intestine, it favours virulence [48]. Moreover, the cAMP-CRP complex also plays a direct regulatory role in the expression of both *intIA* and the *attI* proximal cassettes. Indeed, Pint and Pc are both induced by the binding of the cAMP-CRP complex to the same CRP box located between these two inversely oriented promoters [27] (Figure 2 and 6). It was shown that the intracellular cAMP concentration of *V. cholerae* grown in rich medium increases 2.5-fold between the mid-exponential and early stationary growth phases [49]. This may be responsible for the induction of expression of the first cassettes during entry into stationary phase, which resembles the late stage of infection. This cAMP increment can also explain the induction of *intIA*, through direct activation by the cAMP-CRP complex and also through indirect activation, via a cAMP-dependent induction of the SOS response upon entry in stationary growth phase [44].

Although the extracellular concentration of cAMP increases in the intestinal mucosa at the late stage of infection [41], [42], it seems to have no effect on the formation of the cAMP-CRP complex and its associated regulatory effects in *V. cholerae*. Indeed, we did not observe any induction of the integron platform promoters when external cAMP was added to the growth medium. Thus, the induction of cassette transcription observed when bacteria enter the stationary phase is independent of the cAMP elevation into the lumen of the small intestine.

As previously suggested, the reordering of existing cassettes and the acquisition of exogenous cassettes are both tightly linked to conditions requiring adaptation [27]. In the light of the regulatory mechanisms described here, we propose that the superintegron integrase and the *attI* proximal cassette promoters are both active and allow exogenous DNA integration, transcription of cassettes and selection of cassettes that allow a better fitness of the bacterial cell. Rearrangements and expression of the first cassettes while the bacterium is inside the intestine could increase its chances of survival in its next environment.

## Supporting Information

Figure S1 **Alignment of *V. cholerea* N16961 and *V. mimicus* MB451 Pint – Pc regions, from *intIA* to the *attI* recombination point.** The *intIA* gene sequence is shown in bold italicized characters. Regulator binding site are framed. -10 and -35 promoters boxes are shown in grey. The *attI* recombination point is indicated by a vertical bar.
(TIF)
