# Supplementary figures and images for: The Superintegron Integrase and the Cassette Promoters Are Co-Regulated in Vibrio cholerae
Source: PLoS One. 2014 Mar 10;9(3):e91194. doi: 10.1371/journal.pone.0091194 (PMC3948777; doi:10.1371/journal.pone.0091194)

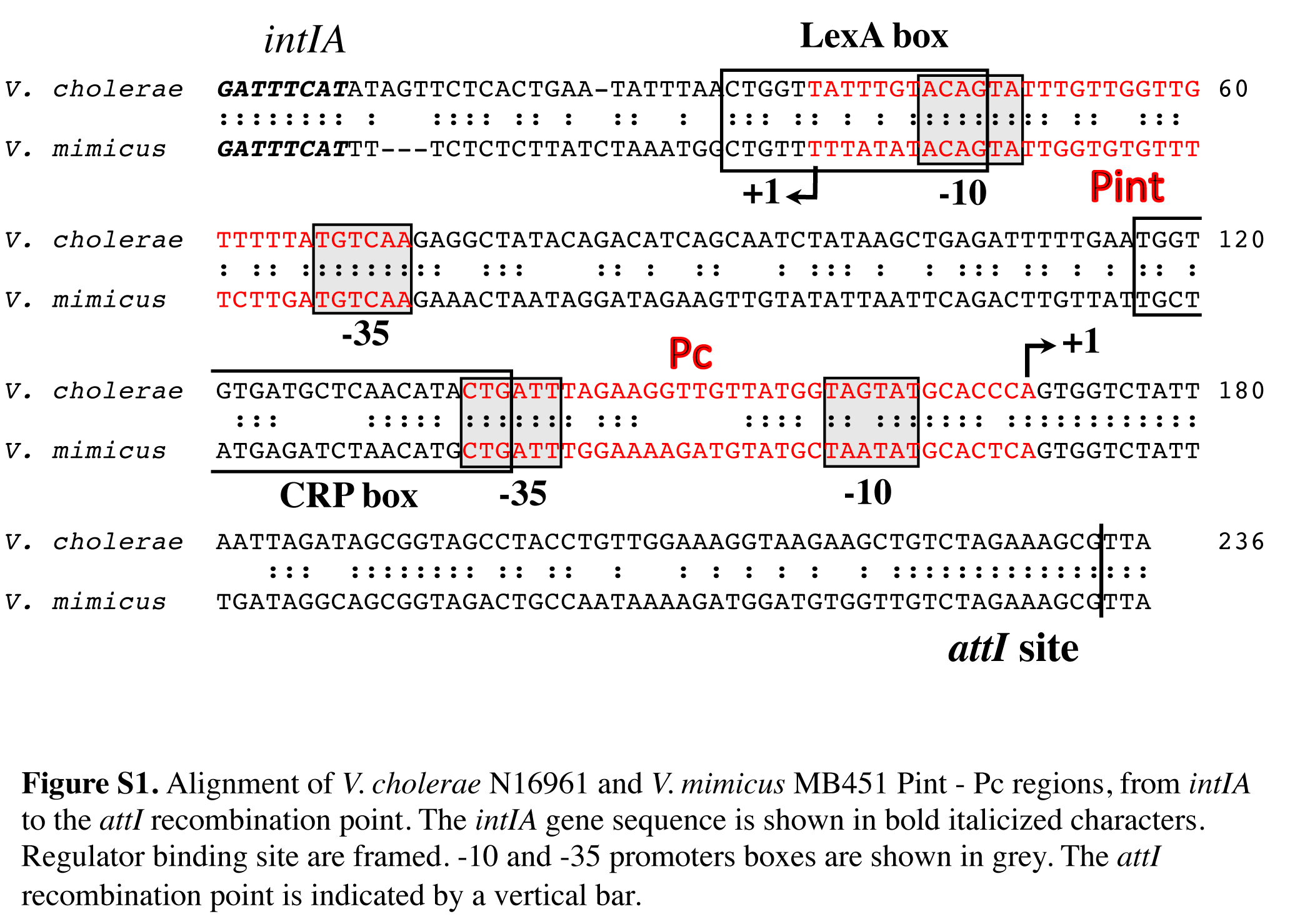

Supplement: Figure S1 — Alignment of V. cholerea N16961 and V. mimicus MB451 Pint – Pc regions, from intIA to the attI recombination point. The intIA gene sequence is shown in bold italicized characters. Regulator binding site are framed. -10 and -35 promoters boxes are shown in grey. The attI recombination point is indicated by a vertical bar. (TIF) [file pone.0091194.s001.tif]
